# Regenerative peripheral nerve interface reduces the incidence of neuroma in the lower limbs after amputation: a retrospective study based on ultrasound

**DOI:** 10.1186/s13018-023-04116-6

**Published:** 2023-08-24

**Authors:** Zhiyu Lin, Ping Yu, Zheng Chen, Guangxue Li

**Affiliations:** 1 Plastic Surgery Department, Peking University People’s Hospital, No. 11 Xizhimen South Street, Xicheng District, Beijing, China; 2 Ultrasound Department, Peking University People’s Hospital, No. 11 Xizhimen South Street, Xicheng District, Beijing, China; 3 Plastic Surgery Department, Peking University Third Hospital, No. 49 North Garden Road, Haidian District, Beijing, China

**Keywords:** Symptomatic neuroma, Regenerative peripheral nerve interface, Ultrasound, Assessment

## Abstract

**Background:**

Amputees suffer from symptomatic neuroma and phantom limb pain. Regenerative peripheral nerve interface (RPNI) has recently been regarded as an effective method to prevent neuroma after amputation. However, the verifications of RPNI efficacy are mostly based on subjective evaluation, lacking objective approaches. This study aims to unveil the effect of RPNI on preventing neuroma formation and provide evidence supporting the efficacy of RPNI based on ultrasound.

**Methods:**

Amputees of lower limb at Peking University People’s Hospital from July 2020 to March 2022 were analyzed retrospectively. The clinical data collected consisted of general information, pathology of primary disease, history of limb-salvage treatment, amputation level of nerve, pain scales such as the Numerical Rating Scale (NRS) and the Manchester Foot Pain and Disability Index (MFPDI). Three months after amputation, the transverse diameter, anteroposterior diameter, and cross-sectional area of neuromas in stump nerves at the end of residual limbs were measured using ultrasound and compared to adjacent normal nerves.

**Results:**

Fourteen patients were enrolled in the study, including 7 in the traditional amputation group (TA group) and 7 in the RPNI group. There was no significant difference in basic information and amputation sites between the two groups. The NRS and MFPDI scores of patients in RPNI group were significantly lower than those in TA group, and decreased with the follow-up time increasing, indicating that RPNI could reduce symptomatic neuroma pain. The comparison of preoperative ultrasound and postoperative pathology showed ultrasound could reflect the size of neuroma in vivo. Independent-sample *t* tests indicated that the ratios of anteroposterior diameter, transverse diameter and area of the cross section of both the neuroma and adjacent normal nerve obtained via ultrasound were significantly reduced in the RPNI group.

**Conclusion:**

This study suggested that RPNI can effectively prevent the formation of symptomatic neuroma after amputation using ultrasound.

## Introduction

The number of amputations increases greatly as a result of work injury, traffic accidents, natural disasters, infections, endocrine diseases and tumors. There are approximately 4 million amputees in China according to the National Bureau of Statistics, which has a certain upward trend. Moreover, the amputations of major limbs can lead to painful and disabling sensory experiences, and phantom limb pain and neuroma pain are typical examples [1]. Approximately 10% of amputees suffer from persistent postamputation pain due to neuroma, which is a common complication after amputation [2–4]. This kind of pain is often continuous and becomes severe when slightly touched. In addition, phantom limb pain is often associated with neuroma. It is characterized by painful sensations perceived in the missing limb after amputation, destroying the ability to wear prostheses and the quality of life [1].

Since both physical and psychological harm are brought by amputations, studies on the prevention of complications have never stopped [5]. The existing treatment methods for neuroma and phantom limb pain include conservative treatment and surgical treatment. Conservative treatment consists of drug, desensitization, massage, radiofrequency ablation, electrical stimulation and acupuncture [6]. For most amputees, surgical treatments mainly cope with the nerve stump, including ligation, burying the nerve in muscle, neurorrhaphy-connecting, capping the nerve, targeted muscle reinnervation and so on [7]. However, these methods show shortcomings in clinical practice [8–11].

The regenerative peripheral nerve interface (RPNI) is involved in the reneuralization of alternative targets and preserves the potential of nerve axons to grow and innervate muscles [12]. Current clinical observations have suggested that RPNI has promising potential to diminish both symptomatic neuromas and phantom limb pain [13–15]. However, most of the existing studies on the efficacy of RPNI are based on subjective symptom scores or questionnaires, which lack convincing objective evidence.

Previous studies have confirmed that ultrasound could be employed for the examination and classification of neuromas with high accuracy [16, 17]. It has important value in preoperative evaluation, surgical planning and therapeutic US-guided procedures [18]. There is no doubt that finding appropriate objective methods to evaluate the occurrence of neuroma after amputation will help further prove the good efficacy of RPNI. However, there is no targeted study based on ultrasound to explore the real ability of RPNI to reduce the incidence of neuromas in amputees to date. This study aims to unveil the effect of RPNI on neuroma formation after amputation through ultrasound and to provide evidence supporting the efficacy of RPNI based on objective examination.

## Materials and methods

This study was conducted in accordance with the World Medical Association Declaration of Helsinki (June 1964) and was approved by the Ethics Committee of Peking University People Hospital (Ethics No. 2021PHB162-001). We retrospectively analyzed 14 patients who underwent lower limbs amputation from July 2020 to March 2022, including 7 with traditional amputation (TA) and 7 with RPNI.

The data collected consisted of general information, pathology of primary disease, history of limb-salvage treatment, amputation level of nerve, and ultrasound results, which contained the anteroposterior diameter, transverse diameter and area of the cross section of both the neuroma and adjacent normal nerve. The postoperative pain of symptomatic neuromas in patients is assessed using the Numerical Rating Scale (NRS, 0-11 points) and the Manchester Foot Pain and Disability Index (MFPDI, 17–51 points), with higher scores on both scales indicating more severe pain [19, 20].

All patients underwent either TA or RPNI using standardized procedures performed by an experienced plastic surgeon. Three months after amputation, ultrasound was used to assess the state of stump nerves at the end of residual limbs. The transverse diameter, anteroposterior diameter, and cross-sectional area of neuromas and adjacent normal nerves were measured. Ultrasound data were independently collected and measured by two experienced ultrasound physicians, and their average values were used. Ultrasound scanning was performed using a 9–18 MHz liner transducer (PLT1005BT; Canon i800, Japan).

Data were analyzed using Statistical Product and Service Solutions software (SPSS, version R26.0.0.2, IBM) and GraphPad Prism (version 9.4.0). Continuous variables are expressed as the mean ± standard deviation, and classified variables are expressed as frequencies and/or percentages. For intergroup parameter comparisons, independent or paired sample t tests were performed for data subject to a normal distribution, and independent or paired sample Mann‒Whitney *U* tests were performed for data not subject to a normal distribution. Count data were tested by the *χ*^2^ test or Fisher’s test. *p* < 0.05 was considered statistically significant.

RPNI operation procedure

**Fig. 1:**
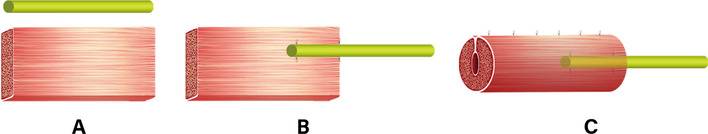
The operation procedure of RPNI. **A** Prepare the stump nerve ends and muscle grafts. **B** Suture the muscle grafts and stump nerve ends. **C** The nerve ends were wrapped by suturing the muscle grafts

All patients’ surgeries are performed by an experienced senior doctor, and the RPNI establishment process is shown in the diagram (Fig. 1).

1. *Preparation for the stump nerve* the proximal end of the limb was dissected, and the stump nerve was completely separated and divided into equal-sized ends of nerve bundles according to the level of the truncated nerve.
2. *Preparation for muscle grafts* Healthy and integrated muscle grafts of approximately 30–15–5 mm were harvested from the amputated limbs.
3. *Construction of RPNI* The separated nerve ends were placed in the muscle grafts along the parallel direction of the muscle fiber, the muscle capsule and the nerve end were sutured with 6–0 nonabsorbable sutures, and all nerve ends were completely wrapped by muscle grafts.
4. Place all the RPNI units away from the surgical incision and the weight-bearing surface of the limbs.

## Results

### Demographic characteristics

A total of 14 patients who underwent amputation surgery at Peking University People Hospital from July 2020 to March 2022 were enrolled, with 7 in the traditional amputation group (TA) and 7 in the regenerative peripheral nerve interface (RPNI) group. The average age was 20.1 ± 13.9 years in the TA and 25.9 ± 18.7 years in the RPNI, without a significant difference (*p* = 0.53). There was no significant difference in the distribution of pathologies of primary diseases, history of limb-salvage treatment, and the amputation levels of nerve between the two groups by Fisher’s exact test (Table 1). In TA, the amputations involved 1 sciatic nerve, 3 tibial nerves and 3 common peroneal nerves, while in RPNI, the amputations involved 3 sciatic nerves, 2 tibial nerves and 2 common peroneal nerves. All patients had no comorbidities, such as hypertension, diabetes mellitus, drinking, smoking and so on.

**Table 1:** The demographic characteristics of the TA group and RPNI group

	TA group (*n* = 7)	RPNI group (*n* = 7)	Fisher’s exact test	*p* value
Age, year	20.1 ± 13.9	25.9 ± 18.7		0.53
Gender, *n* (%)			1	
Male	5 (71.4%)	6 (85.7%)		
Female	2 (28.6%)	1 (14.3%)		
Pathology of primary disease				
Osteosarcoma	6 (85.7%)	5 (71.4%)	1	
Undifferentiated pleomorphicsarcoma	1 (14.3%)	1 (14.3%)		
Fibrosarcoma	0	1 (14.3%)		
History of limb-salvage treatment				
Yes	4 (57.1%)	5 (71.4%)	1	
No	3 (42.9%)	2 (28.6%)		
Amputation level			0.65	
Sciatic nerve	1 (14.3%)	3 (42.9%)		
Tibial nerve	3 (42.9%)	2 (28.6%)		
Common peroneal nerve	(42.9%)	2 (28.6%)		

*TA* traditional amputation, *RPNI* regenerative peripheral nerve interface

### Typical RPNI cases

**Fig. 2:**
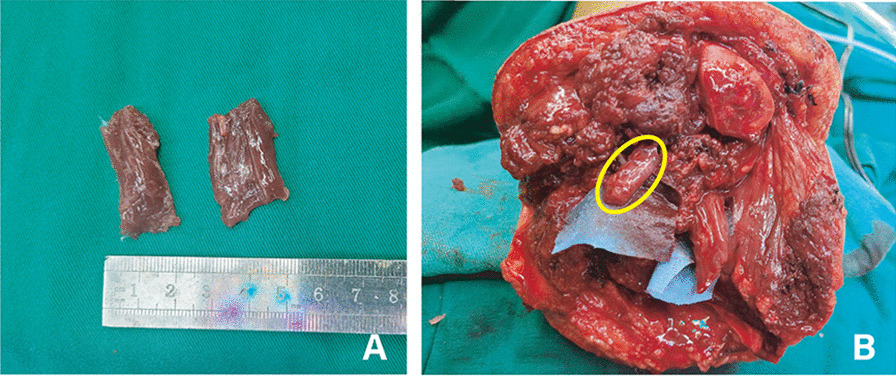
The construction of RPNI in a 17-year-old amputee with fibromatosis. **A** Free muscle grafts obtained from the amputated limb; the size of the muscle grafts was approximately 30–15–5 mm. **B** The yellow circle shows the RPNI built

Figure 2 shows the construction of RPNI in a 17-year-old amputee with fibromatosis in the left leg. The free muscle grafts were obtained from the amputated limb and trimmed to the proper size. Then, the stump nerve ends were finally wrapped by muscle grafts. During the 3-month follow-up, the patients returned to normal life without phantom limb pain or neuroma pain. This condition was maintained for 8 months of follow-up.

### Pain scales

NRS and MFPDI scores were measured at 3 and 6 months in both groups postoperatively. The RPNI patient group demonstrated significantly lower scores at both time points compared to the TA group, with a decreasing trend over time. In contrast, the TA group showed no decrease in scores over time; instead, their scores increased. These findings suggest that RPNI can effectively alleviate postoperative symptomatic neuroma pain and may have a preventive effect on pain during long-term follow-up (Table 2).

**Table 2:** The NRS and MFPDI scores at 3 and 6 months in TA group and RPNI group postoperatively

	TA group (*n* = 7)	RPNI group (*n* = 7)	*p* value
NRS 3 months	6.4 ± 1.1	4.3 ± 1.0	0.002*
NRS 6 months	6.9 ± 1.3	3.1 ± 0.7	< 0.001*
MFPDI 3 months	37.9 ± 2.3	34.7 ± 2.7	0.036*
MFPDI 6 months	39.4 ± 1.1	28.4 ± 1.8	< 0.001*

*TA* traditional amputation, *RPNI* regenerative peripheral nerve interface, *NRS* numerical rating scale, *MFPDI* Manchester foot pain and disability index

*p < 0.05 indicates statistical significance

### Ultrasound result

**Fig. 3:**
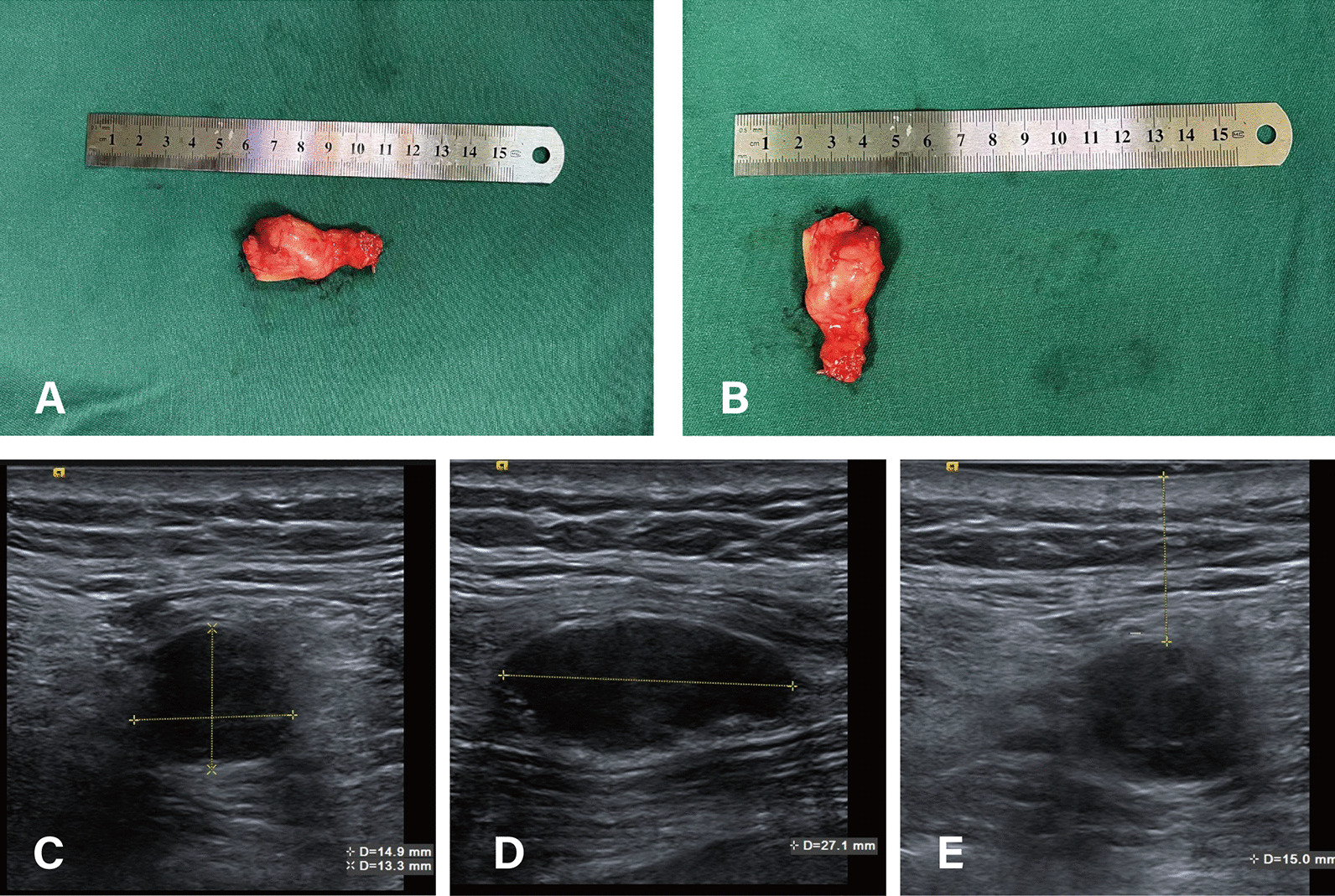
The amputated patient with traumatic neuroma underwent a neuroma ultrasound examination before the surgical resection. **A**–**B** The roughly estimated size of the resected neurofibroma by pathological examination after surgery is about 3 × 2 × 1 cm. **C**–**D** The ultrasound result indicated that a low-echo nodule with a size of approximately 2.7 × 1.5 × 1.3 cm and clear borders could be seen at the far end of the left sciatic nerve stump. **E** The depth of the neuroma from the skin was approximately 1.5 cm

The amputated patient with traumatic neuroma underwent a neuroma ultrasound examination before the surgical resection. The ultrasound result indicated that a low-echo nodule with a size of approximately 2.7 × 1.5 × 1.3 cm and clear borders could be seen at the far end of the left sciatic nerve stump, and no obvious blood flow signal was observed. A low-echo small nodule with a size of approximately 0.5 × 0.3 cm was seen 0.7 cm away from the aforementioned low-echo nodule. The width of sciatic nerve at the proximal 2 cm of the neuroma is about 0.6 cm. The depth of the neuroma from the skin was approximately 1.5 cm. The roughly estimated size of the resected neurofibroma by pathological examination after surgery is about 3 × 2 × 1 cm, which indicates that the results of the ultrasound measurement is consistent with the surgical pathology (Fig. 3).

**Fig. 4:**
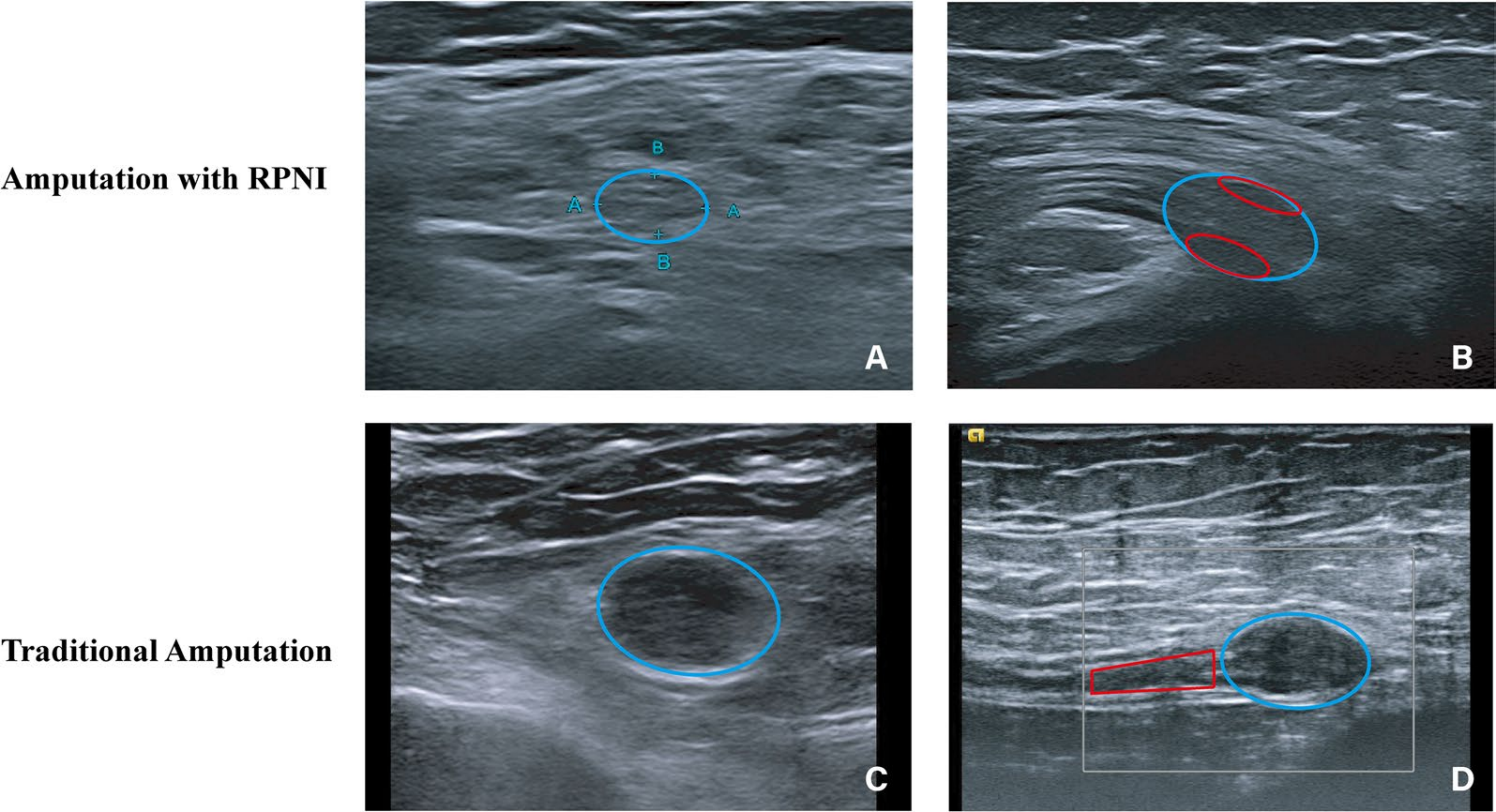
Comparison of the stump nerve end of both the TA group and the RPNI group. Both patients were amputated at the level of the sciatic nerve. **A** The cross section of RPNI (blue circle) was approximately 7.7 × 5.6 mm. The size of the normal nerve adjacent to the distal end was approximately 8.1 × 4.1 mm. **B** Longitudinal section of the RPNI (blue circle). The red circle is the muscle wrapped around the nerve, which was approximately 2.3–2.6 mm in thickness and 8.7 mm in length. **C** The cross section of neuroma in the TA group was approximately 17 × 10 mm (blue circle), and the boundary was clear. **D** Longitudinal section of the neuroma (blue circle). The width of the adjacent normal nerve was approximately 9 mm (red box)

**Fig. 5:**
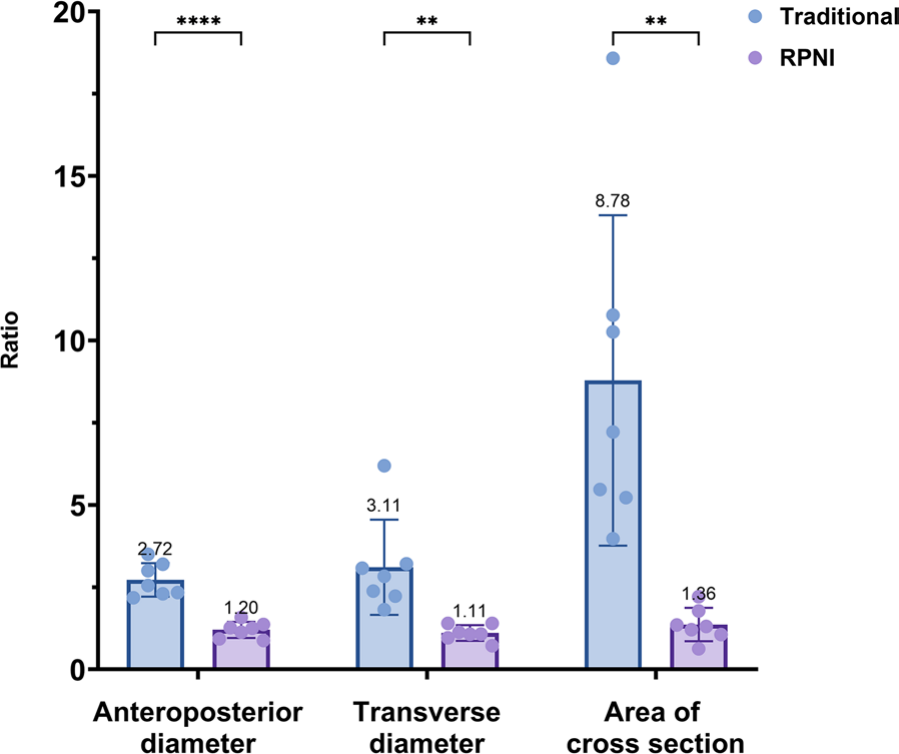
The ratios of the transverse diameter, anteroposterior diameter and the area of the cross section of neuroma and adjacent normal nerve in the TA group and RPNI group. *p < 0.05 indicates statistical significance

A total of 14 stump nerves in 14 patients were examined by high resolution US, including 7 neuromas and 7 RPNI units postamputation. The comparison of the stump nerve ends of both the TA group and RPNI group is shown in Fig. 4. To uniformly assess the enlargement of neuroma at different amputation levels, the transverse diameter, anteroposterior diameter and the area of the cross section of neuroma and adjacent normal nerve were measured, and their ratios were calculated, which were all significantly different (Table 3 and Fig. 5).

**Table 3:** The ratios of the transverse diameter, anteroposterior diameter and the area of the cross section of neuroma and adjacent normal nerve in the TA group and RPNI group

Ratio of neuroma and adjacent normal nerve	TA group	RPNI group	*p* value
Transverse diameter	3.11 ± 1.45	1.11 ± 0.24	0.04*
Anteroposterior diameters	2.73 ± 0.51	1.20 ± 0.25	< 0.001*
Area of cross section	8.78 ± 5.02	1.36 ± 0.51	0.008*

*TA* traditional amputation, *RPNI* regenerative peripheral nerve interface

*p < 0.05 indicates statistical significance

## Discussion

Phantom limb pain and symptomatic neuroma are so common after limb amputation that many patients suffer and are desperate for relief [4]. The suffering of patients not only brings much trouble to the family but is also a heavy burden for the social economy.

Over 100 surgical techniques have been utilized for the prevention and treatment of residual neuromas and phantom limb pain [21]. These approaches can be broadly classified into four categories: closure of the distal nerve end, transposition with implantation, neurorrhaphy, and alternate target reinnervation. All of them have the potential to mitigate the occurrence of neuromas and phantom limb pain to varying extents following amputation, accompanied by their respective merits and drawbacks [22–24]. We believe that the optimal treatment for neuromas should provide targets for axonal regeneration to prevent excessive and disordered growth of the nerve stump axons without causing significant donor site morbidity. Recent research indicates that the regenerative peripheral nerve interface (RPNI) is a promising treatment for neuromas. It can significantly reduce the occurrence of neuroma after amputation by connecting the axon of the nerve stump with a free muscle graft. What’s more, RPNI averts damage to the donor area, circumvents the issue of size disparity at the junction, and harbors the potential to facilitate the transmission of neural signals for motor and sensory prosthesis control [23].

Sofija Pejkova, Kubiak, Woo, Cederna and others found in a series of previous clinical studies that RPNI reduced the probability of symptomatic neuroma pain or phantom limb pain in amputees at different sites and effectively improved the postoperative quality of life, which maintained a stable effect during a long follow-up period [14, 15, 25, 26]. The earlier clinical observation in our center was similar to the studies mentioned above. At the same time, our basic research suggested that RPNI could reduce the incidence and size of neuroma in a rat sciatic nerve amputation model. Pathological sections showed that the density and fibrosis of nerve axons at the stump in the RPNI group were significantly decreased, which partly clarified the related mechanism of RPNI in reducing the occurrence of neuroma [27]. In addition to lessening postoperative complications, RPNI can convert the nerve signals of the stump into muscle signals and theoretically amplify them. These amplified EMG signals can be used to control smart prostheses, which is also beneficial for improving amputees’ quality of life [28].

It was often to evaluate the degree of symptomatic neuroma and phantom limb pain after amputation by summarizing postoperative complications combined with subjective evaluation, including visual analog scale, PROMIS Pain Interference score, the postoperative wearing of prosthesis and the effect on the quality of life [29]. The results of this study demonstrated that patients in the RPNI group had significantly lower NRS and MFPDI scores compared to those in the TA group at both 3- and 6-month post-operation. This suggests that RPNI technology may be effective in reducing and preventing postoperative symptomatic neuroma pain. However, these assessment methods are based on the subjective feelings of patients. Although they effectively illustrate the improvement of RPNI on the subjective feelings of amputees, there is still a lack of objective means to evaluate the preventive effect of RPNI on symptomatic neuroma and phantom pain.

At present, US has been employed in the diagnosis and treatment of neuroma. Anne analyzed the US data of 38 patients with suspected subcutaneous neuroma and further combined these data with the subsequent surgical and pathological data of 13 patients, which verified that US had high accuracy in displaying and classifying the subcutaneous neuroma of both the upper and lower limbs. This demonstrates that ultrasound plays an important role in the preoperative planning and treatment of neuromas [18]. Additionally, Zeidenberg and Koray et al. confirmed the effectiveness of ultrasound in the diagnosis of neuroma [30, 31]. Furthermore, Bianca’s and Xu’s teams compared and analyzed the difference between MRI and ultrasound and found that the ability of ultrasound in the diagnosis of neuroma was even better than MRI [32, 33]. In terms of treatment, US-guided drug injection, radiofrequency ablation and surgical resection all have corresponding effects on neuromas. Ultrasound guidance helps to make the treatment process safer and simpler, reduce posttreatment complications and shorten the recovery time of patients [33–36].

Previous studies have demonstrated that ultrasound, a well-established and accessible technology, can assist surgeons in diagnosing, locating, and treating peripheral nerve disorders., which holds significant potential for widespread clinical application [37]. However, there is no study on the benefit of ultrasound to evaluate the preventive effect of RPNI on symptomatic neuroma after amputation. Therefore, we detected the occurrence of neuroma in the TA group and RPNI group with the aid of ultrasound. There was no significant difference in the distribution of amputation between the two groups. To reduce the heterogeneity of patients and the influence of different amputation levels, we adopted the ratios of the transverse diameter, anteroposterior diameter and the area of the cross section of neuroma and adjacent normal nerve as comparative indexes to assess the true value of RPNI. Our results showed that all three indexes were significantly reduced in the RPNI group compared with the TA group, indicating that RPNI could effectively decrease the occurrence and size of neuroma after amputation. Ultrasound has unique advantages over subjective evaluation methods. First, ultrasound is an objective examination that can directly observe the existence of neuroma, display the stump nerve ends wrapped by RPNI, accurately measure the size of neuroma and obtain the characteristic data of neuroma. All these cannot be realized through subjective evaluation. Second, ultrasound has the advantages of being noninvasive, convenient, low cost and having better patient compliance. We can dynamically monitor the growth of neuroma in a period by increasing the frequency of re-examination, which is conducive to timely disposal. In addition, ultrasound helps to judge the survival of RPNI via the assessment of shape, size and echo of muscle grafts. Thus, we advocate promoting the use of RPNI for amputees and combining subjective evaluation with ultrasound examination for postoperative observation.

To the best of our knowledge, this study is the first to confirm the preventive effect of RPNI on neuroma after amputation based on ultrasound. There are some limitations. First, the number of patients is relatively small for a more convincing conclusion, which needs to be improved. Additionally, limited to the number of patients, we failed to divide different amputation levels into subgroups for a more concrete analysis. Therefore, we will further expand the number of patients and improve the analysis methods in the next stage of the study.

## Conclusion

In conclusion, this study demonstrated that RPNI could effectively reduce the incidence of neuroma after amputation based on ultrasound examination. RPNI significantly decreased the ratios of the transverse diameter, anteroposterior diameter and the area of the neuroma cross section and adjacent normal nerve. Ultrasound will be helpful for the monitoring and evaluation of neuroma and RPNI after amputation.
